# Mesoscale Modeling of Steel Fiber Reinforced Concrete Using Geometric Entity Expansion and Point–Line Topology

**DOI:** 10.3390/ma19081508

**Published:** 2026-04-09

**Authors:** Jutong Li, Lu Zhang, Youkai Li, Chaoqun Sun

**Affiliations:** College of Civil Engineering, Shandong Jiaotong University, Jinan 250357, China; 504004@sdjtu.edu.cn (J.L.); z166374@163.com (L.Z.); 114008@sdjtu.edu.cn (Y.L.)

**Keywords:** excluded-volume effect, mesoscale model, Point–Line Method, interference detection, numerical simulation, damage mechanism(s)

## Abstract

**Highlights:**

- A 2D SFRC mesoscale modeling workflow is developed via Python–Abaqus scripting for batch-reproducible specimen generation.
- A geometric entity expansion strategy prevents unrealistic fiber penetration by accounting for the excluded-volume effect during placement.
- A vector-cross-product Point–Line interference method improves discrimination of fiber–aggregate and fiber–fiber interactions compared with circumscribed-circle screening.

**Abstract:**

Mesoscale modeling provides an efficient and cost-effective approach for investigating the damage mechanisms of fiber-reinforced concrete. To address the physical distortion in conventional models that arises from neglecting the volumetric effect of steel fibers and to construct a more realistic random mesoscale model of steel fiber-reinforced concrete (SFRC), this study proposes an efficient modeling method based on geometric entity expansion and point–line topology. First, polygonal aggregates with diverse morphologies are generated using a polar-coordinate perturbation scheme combined with a convex-hull correction algorithm. Next, abandoning the traditional zero-thickness line-segment assumption, steel fibers are expanded into rectangular entities via rigid-body kinematics to explicitly represent their excluded volume. Furthermore, a vector-cross-product-based Point–Line Method is developed to replace conventional circumscribed-circle screening, enabling accurate discrimination of interference interactions between fiber–aggregate and fiber–fiber pairs. An automated framework—consisting of skeleton placement, entity generation, topological discrimination, and mesh mapping—is implemented through a Python 3.13.9 scripting interface, allowing efficient batch generation of high-content mesoscale models with aggregate area fractions up to 70%. The proposed model is then used to simulate the failure process of SFRC specimens under uniaxial compression and benchmarked against experimental results. The results show that the developed mesoscale model accurately reproduces the nonlinear mechanical response and the strengthening–toughening effects of SFRC, achieving a relative error of only 0.31% in peak stress and a root mean square error (RMSE) as low as 1.70 MPa over the full stress–strain curve. The simulations not only confirm the pronounced strength gain due to steel fiber incorporation (~19.7%), but also reveal, at the mesoscale, the mechanism by which fiber bridging suppresses damage localization, thereby demonstrating the reliability and practical effectiveness of the proposed modeling approach.

## 1. Introduction

Concrete is a three-phase composite material consisting of aggregates, mortar, and the interfacial transition zone (ITZ) between them. The mechanical performance of concrete is directly influenced by aggregate characteristics (e.g., shape, size, and content), mortar strength, and the overall gradation of the mixture. Using numerical simulation tools to investigate concrete meso-structure and to realistically capture the interactions among different phases is of great significance for understanding its mechanical behavior and damage evolution, and it has become a major research focus in recent years.

Constructing random aggregate models that better reflect actual service conditions is fundamental to mesoscale studies of concrete. Numerous studies have been conducted on the generation of random aggregate structures. For instance, Xiong et al. [1], based on a comprehensive assessment of the crack-bridging law and a refinement of fiber-spacing theory, improved the distribution algorithm through Python-based secondary development and proposed a three-dimensional modeling method for basalt fiber-reinforced concrete, in which basalt fibers were equivalently simplified at the mesoscale. Liu et al. [2] performed secondary development of ABAQUS using Python 3.13.9 and proposed a new interference-checking method for polygonal aggregates and steel fibers, thereby improving the efficiency of aggregate placement. Salemi et al. [3] proposed a two-dimensional digital specimen generation method for asphalt concrete by combining image scanning with random aggregate blending.

In mesoscale modeling of random aggregates, existing studies have broadly converged toward two technical routes: (1) a geometry-driven strategy of “geometry construction first, meshing second” and (2) a background-mesh-based strategy of “phase identification and mesh mapping.” The former typically constructs aggregate morphology using random packing, Voronoi/Delaunay tessellation, random polygons/ellipses, and related approaches, and then obtains a multi-phase mesh through geometric Boolean operations and remeshing. Its advantages include high geometric fidelity and straightforward integration with CT-based reconstruction; however, its limitations are also evident—Boolean operations, interfacial refinement, and remeshing are computationally expensive, and both reproducibility and efficiency can be constrained in batch studies and parameter calibration tasks [4,5]. By contrast, the background-mesh (or regular-mesh) approach performs phase identification and property assignment for aggregates/ITZ/mortar directly on a pre-existing mesh, thereby avoiding complex Boolean and remeshing procedures. This makes it better suited for statistically meaningful batch generation, comparative studies, and sensitivity analyses. For example, Sun et al. proposed a high-fidelity and efficient three-dimensional mesoscale modeling algorithm that emphasizes generation efficiency and reusability, providing a useful framework for “engineering-oriented batch modeling” [6].

With respect to mesoscale representation of the “random aggregate–ITZ” system, research has progressed from the question of whether to model the ITZ explicitly to how to reasonably characterize the ITZ thickness and its property gradient. A substantial body of work has shown that the ITZ thickness, its elastic–plastic/damage parameters, and its interfacial behavior with aggregates and mortar can significantly influence macroscopic stiffness, peak strength, and the post-peak softening response, with particularly pronounced sensitivity in crack-dominated loading cases such as tension, splitting, and bending [7,8,9,10]. Building on this, numerical methods for crack propagation have also continued to evolve: beyond conventional damage-plasticity and cohesive-zone models, the phase-field method and phase-field–cohesive-zone coupling (PF-CZM) have been widely applied in mesoscale fracture analyses of concrete-like brittle materials due to their advantages in handling complex crack topologies, branching, and coalescence [11,12]. For instance, Li et al. combined PF-CZM with random aggregate models to simulate complex three-dimensional mesoscale damage and fracture processes, highlighting its potential for controlling mesh sensitivity and capturing crack paths [13].

In mesoscale modeling of fiber-reinforced concrete, existing studies likewise exhibit two mainstream strategies. One is equivalent fiber simplification (e.g., equivalent constitutive behavior, equivalent damage, or equivalent bridging laws), which facilitates engineering applications and parameter calibration. The other is explicit discrete fiber modeling, in which fibers are directly represented by beam/truss/embedded elements to capture fiber geometry and orientation, thereby enabling the description of fiber–matrix bond–slip, pullout, and bridging-induced toughening mechanisms [4,14]. Review studies have shown that, for dynamic loading, impact, and fracture-toughness assessment, an explicit mesoscale description of fiber orientation, dispersion, and spatial relationships with aggregates can substantially enhance the explanatory power for crack patterns, energy dissipation, and ductility improvement [4]. On the other hand, the computational cost and modeling complexity of explicit approaches increase accordingly, particularly when fibers and aggregates are simultaneously discretized and geometric intersection or penetration must be avoided. In such cases, “random placement–interference checking–reproducible generation” becomes a critical technical component [1,15,16].

To address the generation of coupled fiber–aggregate mesoscale configurations, recent studies have incorporated the crack-bridging law and fiber-spacing theory into the modeling workflow, and—together with Python-based secondary development—have improved algorithms for aggregate generation, fiber placement, and fiber–aggregate contact discrimination, thereby establishing more applicable three-dimensional random fiber–aggregate mesoscale finite element frameworks [1]. Meanwhile, for the fracture and tensile response of steel fiber-reinforced concrete (SFRC), discrete and hybrid approaches such as the finite–discrete element method (FDEM) have also been employed to investigate crack evolution and fiber-bridging effects, further strengthening the explanatory link between underlying mechanisms and macroscopic response [15]. In addition to explicit fiber representations, some studies have modeled the failure process of steel fibers from an equivalent-failure perspective, providing a compromise between physical fidelity and computational efficiency to some extent [14].

Although the relevant research base is broad, a clear limitation remains for this line of work. For two-dimensional plane-strain mesoscale models, there is still a lack of a batch-reproducible, “direct mesh mapping” configuration-generation workflow that can simultaneously satisfy the practical engineering requirements of random aggregates + explicit ITZ + explicit steel fibers (T2D2) + prevention of fiber penetration into aggregates + direct writing/editing of the INP file. In the existing literature, available approaches tend to fall into one of three categories: (1) geometry-Boolean operations and remeshing (insufficient efficiency for batch generation), (2) equivalent fiber representations (limited capability to capture discrete fiber effects), or (3) methods that do not provide a readily reusable INP-level automated generation chain [4,5,6,13].

Therefore, coupling background-mesh phase identification with random fiber placement using fast geometric pre-screening followed by precise interference checking, and implementing Abaqus secondary development via Python to enable rapid configuration generation and mechanical response analysis for two-dimensional SFRC mesoscale specimens, can not only support parameter sensitivity and statistical investigations but also provide a more engineering-oriented and practical modeling paradigm for SFRC mesoscale simulations.

Based on the above research background, this study aims to develop an efficient and reproducible two-dimensional mesoscale modeling framework for SFRC that can better preserve geometric realism during random model generation. In conventional line-segment-based fiber placement schemes, the volumetric occupation of fibers is often neglected during geometric generation, which may lead to unrealistic fiber–aggregate or fiber–fiber penetration and reduced configurational fidelity. To address this issue, a geometric entity expansion strategy is introduced to represent fibers as finite-width entities during the generation stage, while a point–line topology-based interference detection method is established to improve the accuracy and efficiency of geometric discrimination.

On this basis, a Python–ABAQUS scripting workflow is developed to integrate skeleton placement, entity generation, topological discrimination, and mesh mapping, enabling batch generation and reproducible construction of SFRC mesoscale models. The primary contribution of this work is therefore at the geometric and computational modeling levels, with the mechanical simulation serving as a validation of the proposed framework under uniaxial compression. Through comparison with experimental results, the study further evaluates the capability of the proposed method to capture the macroscopic response trend and mechanism-consistent mesoscale damage evolution of SFRC within the scope of a two-dimensional idealization.

## 2. Generation Methodology and Algorithms for Random Mesoscale Structures

### 2.1. Random Aggregate Generation

Aggregates play a crucial role in concrete members, particularly in load transfer. In two-dimensional mesoscale random aggregate models, converting a three-dimensional gradation curve (specified in terms of volume or mass fraction) into a two-dimensional sectional gradation (expressed as an area-based probability distribution) is most commonly performed using the stereology-based Walraven equation [17]:

(1) $$\begin{array}{c} P_{c}(D<D_{0})=P_{k}[1.065{\left(\frac{D_{0}}{D_{\max}}\right)}^{1/2}-0.053{\left(\frac{D_{0}}{D_{\max}}\right)}^{4}-0.012{\left(\frac{D_{0}}{D_{\max}}\right)}^{6}\\ -0.0045{\left(\frac{D_{0}}{D_{\max}}\right)}^{8}-0.0025{\left(\frac{D_{0}}{D_{\max}}\right)}^{10}] \end{array}$$

In this equation, $D_{0}$ denotes the current sieve opening size and $D_{\max}$ is the maximum aggregate size. $P_{k}$ represents the three-dimensional volume fraction of coarse aggregates in the specimen. $P_{c}(D<D_{0})$ denotes, on the two-dimensional section, the cumulative probability that a randomly selected point falls within an aggregate whose equivalent sectional diameter is smaller than the sieve opening size $D_{0}$.

Aggregates commonly exhibit irregular polyhedral shapes; therefore, to better represent realistic conditions, irregular polygons are used to construct the aggregate phase in the numerical model. In a two-dimensional plane, generating concrete aggregates as convex polygons provides an effective and computationally efficient way to rapidly create polygonal aggregates. Accordingly, the random aggregate generation procedure adopted in this study is as follows:

#### 2.1.1. Generation of Aggregate Feature Points

Assuming an equivalent aggregate radius of R, the algorithm randomly samples N polar angles $\theta_{i}$ from a uniform distribution in the polar coordinate system. To convert the polar parameters into geometric inputs required for finite element modeling, the feature points $P_{i}$ are mapped to the global coordinate system using the polar-to-Cartesian coordinate transformation matrix, as given in Equation (2):

(2) $$\left\{\begin{array}{l} x_{i}=x_{c}+R\cos\left(\theta_{i}\right)\\ y_{i}=y_{c}+R\sin\left(\theta_{i}\right) \end{array}\right.,\;(\theta_{i}\sim U(0,2\pi ),\;i=1,2,\ldots ,n)$$

**Figure 1:**
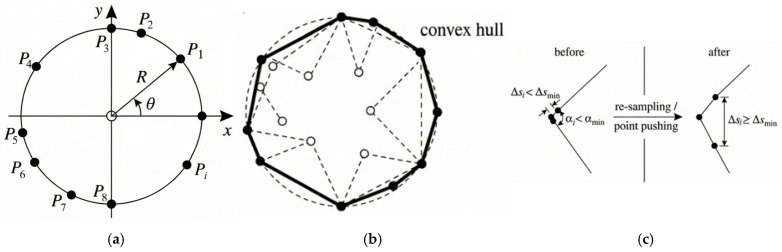
Random aggregate generation process: (**a**) Sampling of feature points in polar coordinates; (**b**) Convexity check and convex-hull correction to reconstruct a convex boundary (interior points are removed); (**c**) Geometric regularity control by re-sampling/point pushing to satisfy minimum edge length and minimum interior-angle constraints.

In Equation (2), $\left(x_{c},y_{c}\right)$ denotes the aggregate centroid coordinates, and N is the number of polygon sides. The procedure is illustrated in Figure 1a.

#### 2.1.2. Convexity Check and Topological Correction

Because the polar angles $\theta_{i}$ are generated in a fully random manner, directly connecting the feature points in their sampling order can readily lead to boundary self-intersections or the formation of concave polygons, which deviates substantially from realistic aggregate geometries. Moreover, concave regions tend to produce highly distorted elements during meshing. Therefore, a strict mechanism for convexity checking and correction is required.

In this study, a vector cross-product method is employed to perform topological validation of the initial polygon. For a counterclockwise-ordered vertex sequence $\left\{P_{1},P_{2},\ldots ,P_{n}\right\}$, the adjacent edge vectors are defined as $e_{i}=P_{i+1}-P_{i}$, and the magnitude of the cross product between adjacent edge vectors is computed as $s_{i}$:

(3) $$s_{i}=e_{i}\times e_{i+1}=(x_{i+1}-x_{i})(y_{i+2}-y_{i+1})-(x_{i+2}-x_{i+1})(y_{i+1}-y_{i})$$

The convexity criterion is defined as follows: if all $s_{i}$ share the same sign, the polygon is identified as convex; if a sign reversal occurs among the $s_{i}$, this indicates the presence of concavity and/or a risk of boundary self-intersection.

For aggregates classified as non-convex, a computational-geometry-based convex hull algorithm is introduced for correction (as shown in Figure 1b). This step automatically removes interior points that cause concavity and reconstructs the outermost boundary connections, thereby ensuring that the generated aggregates exhibit robust geometric convexity and improved numerical stability in subsequent simulations.

#### 2.1.3. Geometric Regularity Control and Mesh Quality Optimization

Although convexity correction guarantees topological validity, under extreme random sampling, adjacent feature points may become excessively close, resulting in very short edges or extremely sharp angles. Such geometric distortions can lead to locally over-refined meshes, negative Jacobian determinants, or severely distorted aspect ratios during finite element meshing, thereby inducing non-physical stress concentrations and even causing non-convergence. To address this issue, a geometric regularity control mechanism based on dual-threshold constraints is established, as illustrated in Figure 1c. It consists of the following three components:

(1) Minimum edge-length constraint $\left(\Delta s_{\min}\right)$: The Euclidean distance $\Delta s_{i}$ between any two adjacent vertices $P_{i}$ and $P_{i+1}$ along the aggregate boundary is calculated. If $\Delta s_{i}<\Delta s_{\min}$, the vertices are considered excessively clustered locally. The algorithm then automatically performs re-sampling or vertex merging to increase the spacing until the constraint $\Delta s_{i}\geq \Delta s_{\min}$ is satisfied. Here,
  (4) $$\Delta s_{i}=\sqrt{{\left(x_{i+1}-x_{i}\right)}^{2}+{\left(y_{i+1}-y_{i}\right)}^{2}}\geq \Delta s_{\min}$$
(2) Minimum interior-angle constraint $\left(\alpha_{\min}\right)$: To avoid sharp-corner effects, the interior angle $\alpha_{i}$ at each vertex is evaluated. If $\alpha_{i}<\alpha_{\min}$, a small perturbation is applied to that vertex in either the radial or tangential direction to blunt the acute-angle feature.
(3) Iterative optimization: The above correction procedures are implemented within an iterative loop. If the generated aggregate still fails to satisfy the geometric constraints after $N_{\max}$ iterations, the sample is discarded and regenerated. This mechanism preserves aggregate randomness to the greatest extent possible while ensuring mesh quality from the geometric source, thereby markedly improving the convergence and stability of nonlinear simulations.

After generating the aggregate boundary, mesh quality is improved through a lightweight regularization procedure before the final mesh mapping. Specifically, (1) a minimum edge-length constraint is enforced by re-sampling/point pushing to eliminate extremely short edges; (2) a minimum interior-angle constraint is applied to avoid sharp corners that may lead to highly distorted elements; and (3) local geometric regularization is performed iteratively until the polygon satisfies the prescribed thresholds. These steps aim to reduce element distortion (e.g., highly skewed or sliver elements) after phase identification and to improve the robustness of the subsequent finite-element analysis.

### 2.2. Random Generation of Steel Fibers

As a high-modulus, rigid inclusion, the spatial distribution of steel fibers within the concrete matrix directly governs the strengthening and toughening mechanisms of the composite. Unlike flexible fibers, which may be idealized as zero-thickness line segments, steel fibers possess a well-defined geometric shape. To overcome the limitations of conventional models that neglect the excluded-volume effect of fibers, this study proposes a parametric modeling strategy based on “center placement–random orientation–entity expansion.”

Parametric strategy for random fiber generation. Random fibers are generated using a “center placement–random orientation–entity expansion” strategy. First, fiber centers are sampled uniformly within the admissible domain, and a preliminary fiber skeleton is represented by a zero-thickness line segment with length $L_{f}$. Second, a random orientation angle θ is assigned for isotropic distribution), yielding the fiber direction vector. Third, the fiber entity is obtained by expanding the centerline into a finite-width rectangle of thickness $d_{f}$ using rigid-body kinematics, which explicitly accounts for the excluded-volume effect during placement. The generated fiber entity is then subjected to a two-stage interference check (fast screening followed by precise point–line discrimination) to ensure non-penetration with aggregates and other fibers.

#### 2.2.1. Random Parameterization of In-Plane Position

Under a two-dimensional plane-strain condition, the initial state of a steel fiber is uniquely determined by its spatial pose, defined by the centroid location and orientation angle. Let the placement domain be Ω, and let the geometric parameters of a single fiber be its length $L_{f}$ and diameter $d_{f}$.

**Figure 2:**
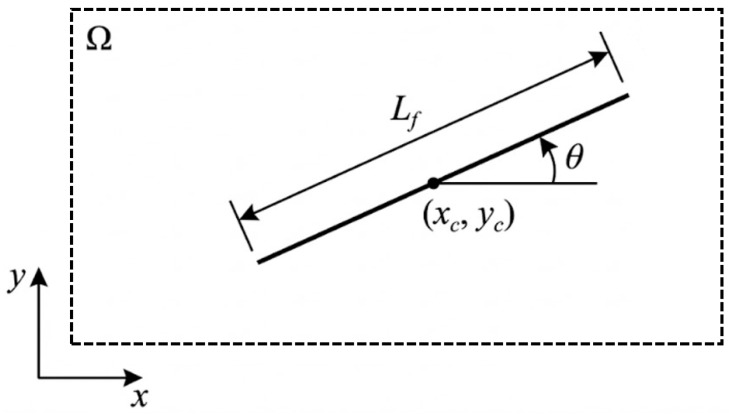
Schematic illustration of steel fiber.

As shown in Figure 2, the algorithm first generates the fiber centroid coordinates $\left(x_{c},y_{c}\right)$ within the placement domain using a Monte Carlo scheme and then assigns a random orientation angle $\theta_{c}$. To mimic the stochastic distribution of steel fibers during casting, the above parameters are assumed to follow uniform distributions, as given in Equation (5):

(5) $$\left\{\begin{array}{l} x_{c}\sim \left(0,W\right)\\ y_{c}\sim \left(0,H\right)\\ \theta_{c}\sim \left(-\pi ,\pi \right) \end{array}\right.$$

In Equation (5), W and H denote the specimen width and height, respectively.

A “generate first, trim later” strategy is adopted, allowing fibers to be initially placed near the boundaries; any portions extending outside the domain are subsequently removed via Boolean trimming. This treatment helps maintain a uniform fiber distribution in the boundary regions.

#### 2.2.2. Geometric Entity Expansion Based on Rigid-Body Kinematics

To explicitly account for the physical volume of steel fibers during placement, the algorithm abandons the conventional line-segment representation and instead adopts a geometric entity expansion strategy. Specifically, after generating the fiber centerline segment as described in the previous section, the segment is further expanded in space to form a finite-sized geometric entity, thereby better reflecting engineering reality.

The steel fiber is treated as a rigid rectangular entity in the two-dimensional plane, with the major axis denoted by $L_{s}$ and the minor axis denoted by $d_{s}$. Based on rigid-body kinematics, the fiber geometry is obtained via vertex transformation in a local coordinate system. Let the four vertices of the fiber in the local coordinate system be $\left(\pm L_{s}/2,\;\pm d_{s}/2\right)$; then the vertex-coordinate matrix in the global coordinate system, $P_{vertex}$, can be computed using the rotation–translation transformation matrix as follows:

(6) $$\left[\begin{array}{l} x_{i}\\ y_{i} \end{array}\right]=\left[\begin{array}{cc} \cos\theta & -\sin\theta \\ \sin\theta & \cos\theta \end{array}\right]\left[\begin{array}{c} \pm L_{s}/2\\ \pm d_{s}/2 \end{array}\right]+\left[\begin{array}{c} x_{c}\\ y_{c} \end{array}\right],\;\left(i=1,2,3,4\right)$$

Through the above transformation, each generated steel fiber in the numerical model is no longer an idealized zero-thickness mathematical line segment, but a two-dimensional solid entity $\Omega_{f}$ that occupies real physical space.

#### 2.2.3. Equivalent Mapping Mechanism for the Fiber Constitutive Model

Although an entity-based representation is adopted during geometric generation to ensure placement accuracy, a geometry–mechanics decoupling strategy is employed in the finite element solution stage to balance computational efficiency and model scale.

After meshing is completed, the solid fiber entity is reduced to an embedded truss element, and its mechanical behavior is compensated through an equivalent constitutive mapping.

### 2.3. Random Generation of Concrete Constituents

When an external load is applied to concrete, the load is first dispersed through the mortar and then transferred to the coarse aggregates through the weaker Interfacial Transition Zone (ITZ), thereby forming an overall load-bearing skeleton.

#### 2.3.1. ITZ

The ITZ is a porous, relatively weakened yet comparatively stable transition layer located between coarse aggregates and the mortar matrix. Its mechanical properties lie between those of the aggregates and the mortar, and it gradually transitions into the mortar matrix in space. It is generally accepted that larger aggregate sizes tend to form a thicker ITZ due to the effects of hydration and bleeding. To represent this size effect in the mesoscale model, a particle-size-dependent ITZ thickness control rule is introduced. For the i-th aggregate, its equivalent particle size is defined as $D_{i}=\sqrt{4S_{i}/\pi }$, where $S_{i}$ is the area of the *i*-th aggregate polygon in the 2D model, and the ITZ thickness can be expressed as follows:

(7) $$t_{ITZ,i}=t_{0}{\left(\frac{D_{i}}{D_{0}}\right)}^{k}$$

Here, $t_{0}$ is the baseline thickness, $D_{0}$ is the reference particle size, and k is the scale factor (k>0, a larger particle size corresponds to a thicker ITZ). This power-law scaling is introduced as an engineering-oriented, size-dependent assumption to reflect experimentally observed trends that ITZ thickness tends to increase with coarse aggregate size due to the wall effect and local packing characteristics around aggregates [18]. In mesoscale modeling practice, the ITZ thickness is typically on the order of tens of micrometers and is commonly assumed within 20–100 μm [19].

In the validation case of this study, a constant ITZ thickness of 100 μm was adopted for calibration consistency and computational control, while Equation (7) is retained for extended parametric analyses involving particle-size-dependent ITZ thickness.

**Figure 3:**
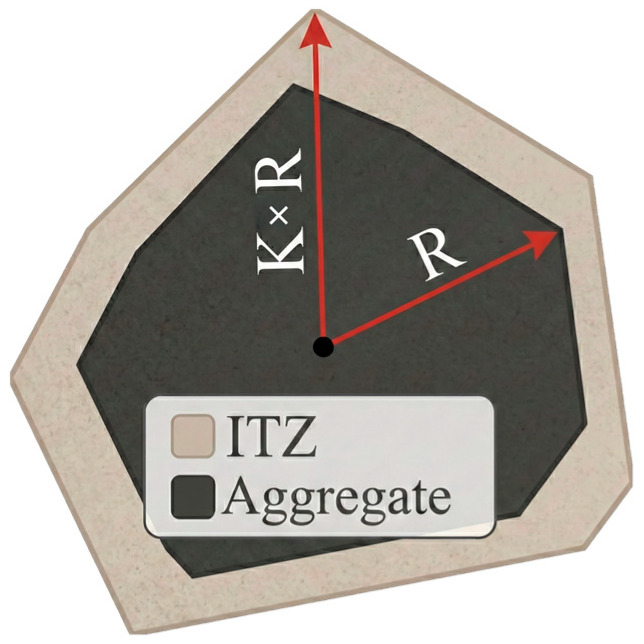
Details of the geometric model of ITZ.

In two dimensions, the ITZ is treated as a thin annular band outside the aggregate boundary. Taking the aggregate polygon as the inner boundary, the ITZ is generated by offsetting the boundary outward along the local outward normal to obtain a transition zone with thickness $t_{ITZ,i}$, thereby achieving a three-phase partition of aggregate–ITZ–mortar (as shown in Figure 3).

#### 2.3.2. Aggregate Area

**Figure 4:**
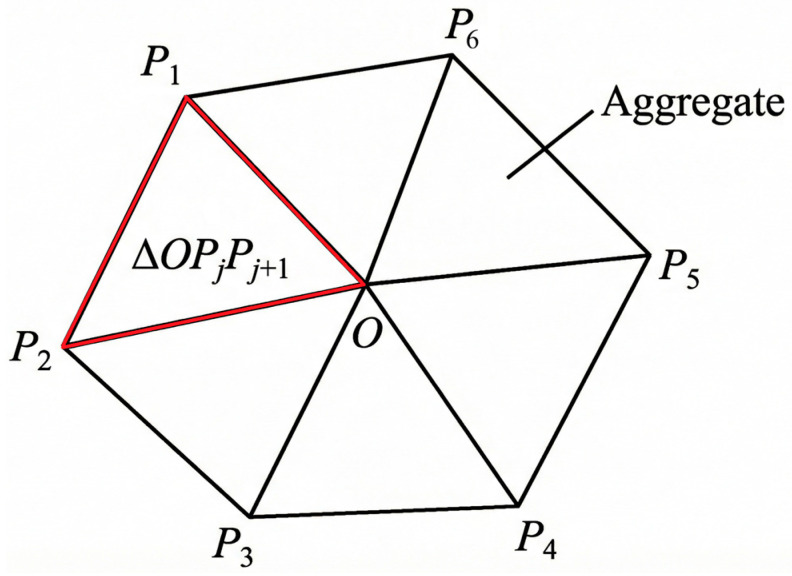
Details of the geometric model of aggregates.

To control the aggregate content, the planar model requires the area of randomly generated aggregates to be calculated. Let the i-th aggregate be a counterclockwise convex polygon $\left\{P_{1},P_{2},\ldots ,P_{n}\right\}$. An interior point O (the circumcenter) is selected, and the polygon is partitioned into triangles $\Delta OP_{j}P_{j+1}(P_{n+1}=P_{1})$. The aggregate area is then obtained by summing the triangle areas, which enables control of the aggregate area fraction (Figure 4). Subsequently, to prevent fibers from intruding into the aggregates/ITZ, fiber–aggregate interference constraints and a fast screening strategy are introduced.

#### 2.3.3. Fiber–Aggregate Contact Determination

After obtaining the fiber endpoints, it is necessary to determine whether the fiber intrudes into the aggregate/ITZ. To improve efficiency, a fast screening step is first performed based on the aggregate circumcircle. Specifically, the shortest distance d from the fiber segment to the circumcenter O is computed. If $d>R_{i}+\delta +t_{ITZ,i}$, the fiber is considered non-intersecting; otherwise, the fiber is rejected or forwarded to an exact intersection check. Where, d denotes the steel fiber diameter. $R_{i}$ is the circumscribed-circle radius of the *i*-th aggregate, used in the circumscribed-circle-based fast screening. δ is a user-defined safety margin to avoid numerical overlap in the fast screening criterion. $t_{ITZ}$ denotes the ITZ thickness.

Let the fiber be a line segment AB with endpoints $A(x_{A},y_{A})$ and $B(x_{B},y_{B})$, and define the vectors as follows:

(8) $$u=\vec{AB}=B-A,v=\vec{AO}=O-A$$

Project v onto u to obtain the projection parameter ξ:

(9) $$\xi =\frac{u\cdot v}{u\cdot u}$$

Note that $u\cdot u={\left\|u\right\|}^{2}$ is the squared length of segment AB.

**Figure 5:**
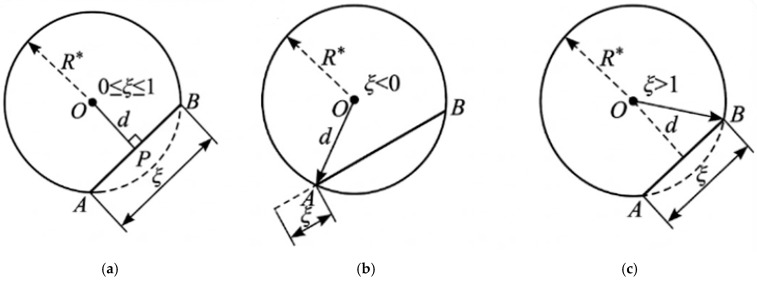
Schematic of the circumscribed-circle–based fast screening for fiber–aggregate interference. (**a**) 0≤ξ≤1: the projection point P lies on segment AB and the minimum distance is OP. (**b**) ξ<0: the closest point is A and the minimum distance is OA. (**c**) ξ>1: the closest point is B and the minimum distance is OB. Here, ξ is the projection parameter defined in Equation (9), d denotes the minimum distance from O to the fiber centerline segment AB, and R* is the circumscribed-circle radius used in the fast screening criterion.

Depending on the location of the projection point on the line, the shortest distance d can be classified into three cases (as shown in Figure 5):

(1) When 0≤ξ≤1, the projection point lies within the segment, and the shortest distance is:

(10) $$d=\left\|v-\xi u\right\|$$

In the fast screening stage, the minimum distance is computed from the fiber centerline to the aggregate circumcenter; the finite fiber thickness is incorporated through the exclusion threshold in Equation (13) by adding d/2 (together with ITZ thickness and safety margin).

(2) When ξ<0, the shortest distance is determined by endpoint A:

(11) $$d=\left\|O-A\right\|$$

(3) When ξ>1, the shortest distance is determined by endpoint B:

(12) $$d=\left\|O-B\right\|$$

Considering the safety margin Δ and the influence of the ITZ thickness $t_{ITZ}$ on the effective exclusion distance, the decision threshold is defined as:

(13) $$R_{i}^{*}=R_{i}+t_{ITZ}+\Delta$$

If d is smaller than the decision threshold, the fiber is considered to have a potential intrusion risk into the aggregate (or its ITZ) and should be rejected or re-sampled in terms of its position/orientation; otherwise, the fiber is deemed to have passed the fast screening step.

## 3. ABAQUS Model Development

To verify the effectiveness of the proposed random aggregate and random steel fiber generation methods, three two-dimensional mesoscale models with dimensions of 150 mm × 150 mm were established, with an average aggregate placement ratio of 42.3% and a steel fiber volume fraction of 1%. The damage and failure processes of these models under uniaxial quasi-static compression were then investigated. All simulations were performed using Abaqus 2022 with Python scripting for model pre-processing [20].

### 3.1. Modeling Assumptions and Their Implications

Aggregates are treated as linear elastic because, under the considered compressive loading level, damage and inelastic deformation are expected to localize primarily within the mortar matrix and the ITZ, while aggregates remain relatively intact compared with the surrounding phases. The ITZ is represented as a weakened mortar phase using a commonly adopted reduction range (70–90%) to reflect its higher porosity and lower stiffness/strength than the bulk mortar [21]. For steel fibers, the fiber–matrix interaction is simplified through an equivalent embedding and mapping strategy to achieve a practical balance between geometric realism during generation and computational efficiency during solving.

These simplifications may influence the quantitative response, especially in the late post-peak regime: (1) neglecting aggregate fracture may slightly affect mesoscale stress redistribution and stiffness evolution; (2) the selected ITZ weakening level affects the onset and extent of damage localization and thus post-peak softening; and (3) the equivalent fiber representation may under-resolve detailed pull-out and anchorage mechanisms, which can contribute to discrepancies in residual strength. Nevertheless, within the scope of the present 2D idealization, these assumptions enable robust batch modeling and mechanism-consistent comparison between plain concrete and SFRC specimens under unified numerical settings.

### 3.2. Material Properties

To describe the mechanical response mechanisms of the mortar, ITZ, aggregates, and fibers, the Concrete Damaged Plasticity (CDP) model was adopted for the mortar and ITZ. The aggregates were simplified as linear elastic, while the fibers were modeled as embedded T2D2 truss elements with a linear elastic axial constitutive law. The fiber–matrix interaction was represented using the embedded constraint together with the adopted equivalent mapping strategy.

#### 3.2.1. Mortar

The CDP model was originally proposed by Lubliner [22] and later extended by Lee and Fenves [23] to represent the coupled plasticity–damage behavior of concrete-like materials. In this study, the CDP parameters for the mortar phase were determined based on calculations in accordance with GB 50010-2010 [24].

#### 3.2.2. Interfacial Transition Zone

The ITZ was modeled as a weakened mortar phase, and its stiffness/strength-related parameters were implemented by reducing the elastic modulus, strength parameters, and damage evolution curves to 70–90% of the mortar values (Table 1), consistent with common mesoscale practice [18]. The ITZ thickness was set to 100 μm for the validation case in this study. Although the proposed framework supports particle-size-dependent ITZ thickness, a constant thickness was adopted here to ensure calibration consistency and computational control.

#### 3.2.3. Aggregates

The specimens had a standard size of 150 mm × 150 mm and employed a two-size gradation for the coarse aggregates, namely 5–10 mm and 10–20 mm. In the two-dimensional mesoscale model, the aggregate phase exhibits substantially higher stiffness and strength than the mortar and ITZ, and damage near the compressive peak is more likely to develop within the matrix and along the interfaces. To reduce computational cost and to highlight the underlying mechanisms associated with the matrix–interface–fiber system, the aggregate phase was simplified as linear elastic, with neither damage nor plasticity considered.

#### 3.2.4. Steel Fibers

**Figure 6:**
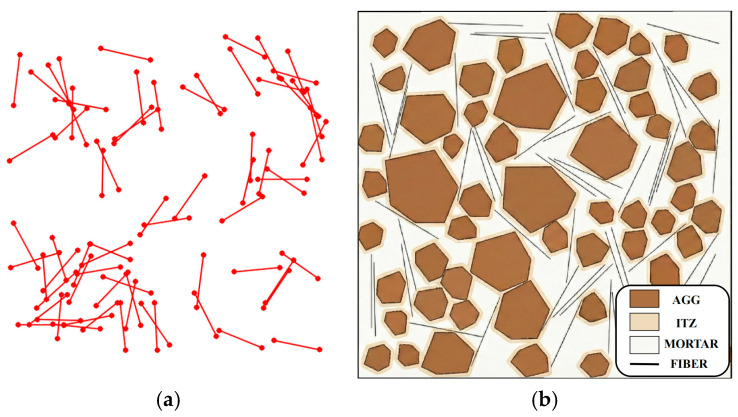
Schematic of model construction: (**a**) Schematic of fiber construction (T2D2 truss elements); (**b**) Schematic of the four-phase finite element model.

The steel fibers used in this study were cold-drawn hooked-end steel fibers, manufactured from high-strength carbon steel. The fiber length was set to 25–35 mm, the diameter was 0.55 mm, and the aspect ratio was 64. The fibers were modeled using T2D2 truss elements and embedded within the matrix to enforce deformation compatibility coupling. The final model is shown in Figure 6b, and the key parameters are summarized in Table 1.

#### 3.2.5. Parameter Determination and Calibration Strategy

To clarify the parameter source and calibration logic, the material parameters used in this study were determined according to a unified procedure. The mortar-phase parameters were established based on the available material information (manufacturer-provided properties and code-based estimates), and then implemented in the CDP framework. The ITZ was treated as a weakened mortar phase using commonly adopted reduction ratios reported in the literature (typically 70–90% of the mortar-related parameters). The steel fiber parameters were defined from the corresponding material properties, and the fiber–matrix interaction was represented through the adopted equivalent embedding and mapping strategy. Detailed parameter values are listed in Table 1 and the corresponding subsections.

It should be emphasized that the matrix-phase parameters (mortar and ITZ) used in the SFRC simulations were not re-calibrated against the SFRC stress–strain response. The same parameter set determined for the plain concrete matrix system was retained, and the SFRC response was obtained by introducing the fiber phase and the corresponding interaction representation. Therefore, the SFRC simulation results reported herein should be interpreted as a parameter-transfer validation under a fixed matrix parameter framework rather than an independent curve-fitting process.

### 3.3. Boundary Conditions and Meshing

**Figure 7:**
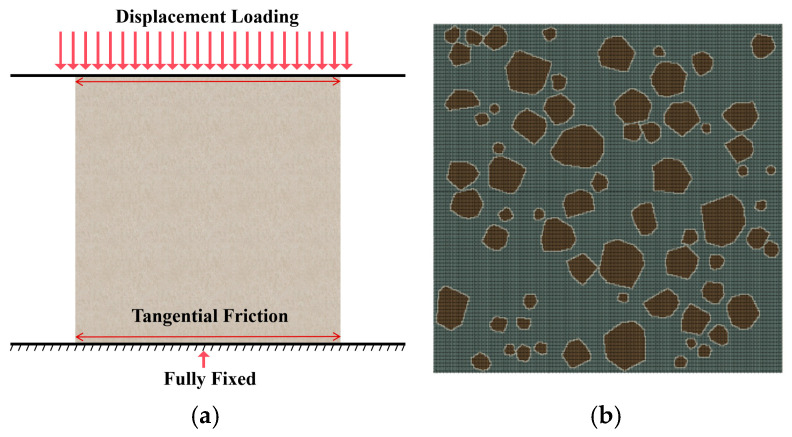
Schematic of model conditions and meshing: (**a**) Loading conditions; (**b**) Meshing.

Following standard compression testing practice, quasi-static displacement control was applied (Figure 7a). The specimen contacted rigid line platens at the top and bottom. Tangential contact was modeled with a penalty-based friction law μ=0.3. The upper platen was prescribed a vertical compressive displacement with all other degrees of freedom constrained, while the lower platen was fully fixed to prevent rigid-body motion.

The mortar, aggregates, and ITZ were discretized using CPE4R plane-strain elements. A uniform mesh size of 0.1 mm was adopted to balance accuracy and efficiency (Figure 7b), which corresponds to approximately 2.25 × 10^6^ plane-strain elements and 2.253 × 10^6^ nodes for the 150 mm × 150 mm specimen.

### 3.4. Contact Properties

The mesoscale model consists of four phases (aggregates, mortar, ITZ, and steel fibers). The aggregate–ITZ–mortar system is represented as conforming partitions on the same background mesh; therefore, no explicit contact algorithm is introduced between these phases, and inter-phase continuity is ensured by the shared mesh topology.

**Figure 8:**
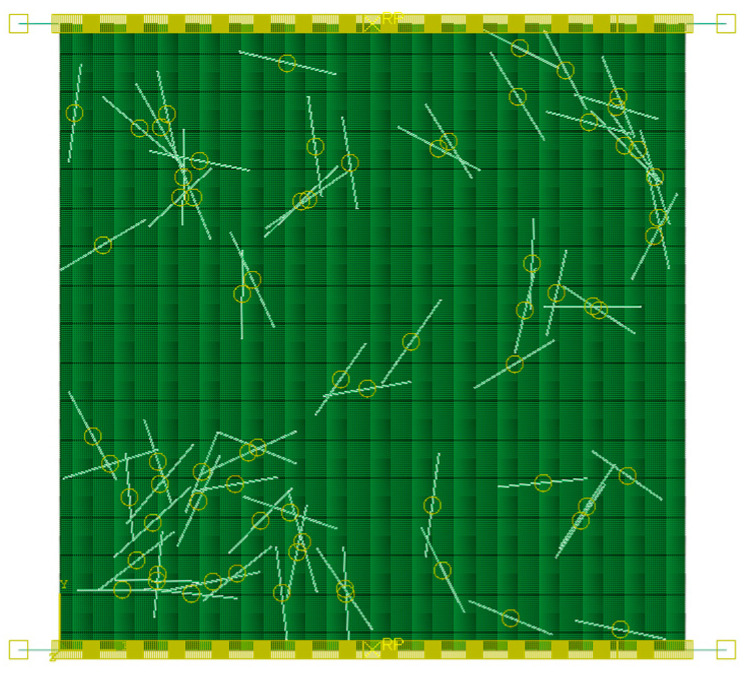
Schematic diagram of interaction setup.

Steel fibers are modeled as embedded T2D2 truss elements, and the fiber–matrix coupling is enforced through the embedded constraint (deformation compatibility) together with the adopted equivalent mapping strategy. In the present study, an explicit bond–slip law is not introduced. The interaction between the rigid platens and the specimen is modeled as surface-to-surface frictional contact with hard normal contact and Coulomb friction tangentially, as illustrated in Figure 8.

## 4. Validation and Analysis of Numerical Results

### 4.1. Comparison of Macroscopic Load-Carrying Behavior

**Figure 9:**
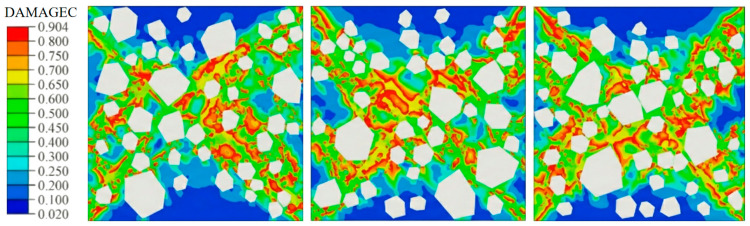
Damage contour plot of plain concrete.

**Figure 10:**
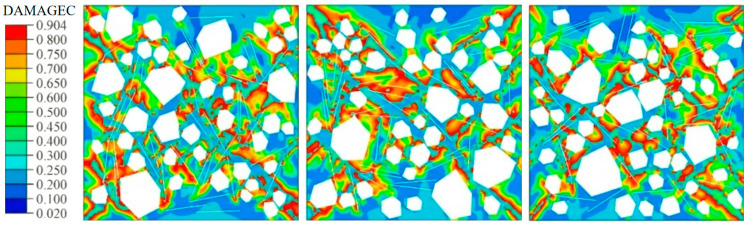
Damage contour plot of steel fiber reinforced concrete.

Figure 9 and Figure 10 compare the damage contours of three specimen groups under uniaxial compression, evaluated using the CDP compressive damage variable DAMAGEC. Plain concrete tends to develop a through-going, shear-dominated failure band after peak load, with damage concentrated in a narrow zone indicative of brittle instability. By contrast, steel fiber-reinforced concrete shows more diffuse damage and a delayed dominant band. Elevated fiber axial stress near the main failure zone indicates effective bridging, which constrains crack growth and dissipates energy, thereby suppressing damage localization and delaying through-crack formation.

### 4.2. Error Analysis and Reliability Assessment

To systematically evaluate the fidelity of the proposed mesoscale model in predicting the mechanical behavior of steel fiber-reinforced concrete, three metrics were selected: peak stress, peak strain, and goodness-of-fit over the full stress–strain curve.

#### 4.2.1. Definition of Evaluation Metrics

To quantify the discrepancy between the numerical predictions and experimental data, three statistical indicators were introduced. First, for key characteristic points such as the peak load, the relative error δ was used, defined as follows:

(14) $$\delta =\frac{\left|V_{\exp}-V_{sim}\right|}{V_{\exp}}\times 100\%$$

Here, $V_{\exp}$ denotes the characteristic value measured from the experiments, and $V_{sim}$ denotes the corresponding value obtained from the numerical simulation.

In addition, to evaluate the overall agreement of the full stress–strain curve, the root mean square error (RMSE) and the mean absolute percentage error (MAPE) were introduced. These two metrics effectively reflect the stability of the model predictions over the entire loading history, as defined below:

(15) $$RMSE=\sqrt{\frac{1}{N}\sum_{i=1}^{N}{\left(\sigma_{\exp,i}-\sigma_{sim,i}\right)}^{2}}$$

(16) $$MAPE=\frac{1}{N}\sum_{i=1}^{N}\left|\frac{\sigma_{\exp,i}-\sigma_{sim,i}}{\sigma_{\exp,i}}\right|\times 100\%$$

Here, N is the total number of sampling points; $\sigma_{exp,i}$ is the stress at the i-th sampling point on the experimental curve; and $\sigma_{sim,i}$ is the corresponding stress on the simulated curve.

#### 4.2.2. Comparison of Peak Characteristics

Peak load represents the ultimate load-carrying capacity of the material prior to failure and is a primary indicator for validating the proposed mesoscale model. Based on the above metrics, Table 2 summarizes the comparisons between the experimental results and the numerical predictions at the key characteristic points.

Statistical analyses indicate that the proposed mesoscale modeling strategy based on geometric entity expansion achieves high fidelity in predicting ultimate strength:

(1) Plain concrete (PC) control group: The experimentally measured mean peak stress was 41.51 MPa, while the numerical prediction was 41.32 MPa. The two values are in excellent agreement, with a relative error δ of only 0.46%. This confirms the accuracy of the polygonal aggregate generation algorithm and the calibration of the mortar/ITZ constitutive parameters, which together capture the initial load-bearing skeleton of the matrix.
(2) SFRC group: With steel fiber incorporation, the experimental peak stress increased markedly to 49.69 MPa, indicating a clear strengthening effect. The numerical model captured this increase well, predicting a peak stress of 49.54 MPa, with an even smaller relative error of 0.31%.

Mechanistic consistency: Comparing PC and SFRC shows that steel fibers increased the compressive strength by approximately 19.7% (from 41.51 MPa to 49.69 MPa). Beyond achieving a low absolute prediction error (<0.5%), the model also reproduced the magnitude of this nearly 20% strength gain. These results substantiate that the proposed fiber solidification modeling and Point–Line topological discrimination can realistically construct the spatial fiber skeleton within the matrix, thereby enabling the strengthening and crack-arrest mechanisms of fibers to be accurately activated in the numerical simulations.

#### 4.2.3. Full-Curve Evolution and Damage Mechanism Analysis

It should be noted that in a typical uniaxial compression test, the upper platen is often seated with a spherical hinge (or a seated block), allowing limited rotation and reducing end restraint. In the present numerical model, rigid platens with contact were adopted to ensure computational stability and reproducible batch analyses. This boundary idealization may influence the local stress redistribution near the specimen ends and, consequently, the peak strain and the post-peak localization pattern. Therefore, the experimental–numerical comparison in this study focuses primarily on the macroscopic stress–strain response trend and the mechanism-consistent damage evolution within the scope of the adopted boundary setting.

**Figure 11:**
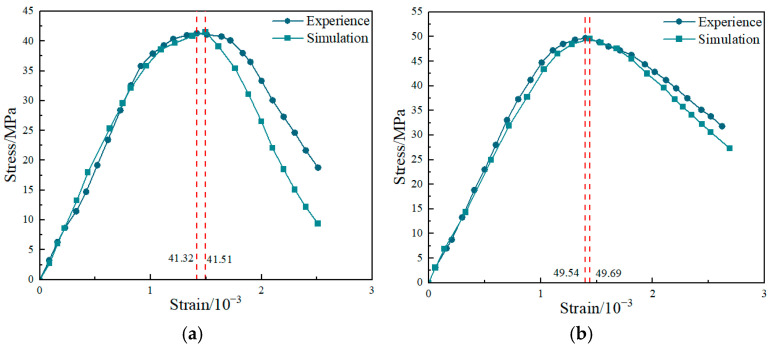
Comparison of stress–strain curves, (**a**) Plain concrete, (**b**) Fiber-reinforced concrete.

Figure 11 compares the experimental and numerical stress–strain curves of plain concrete and steel fiber-reinforced concrete. Overall, the numerical results reproduce the elastoplastic evolution and post-peak load-carrying response of both specimen types, and the predicted curve shapes agree well with the experimental trends.

Based on the full stress–strain curve comparison in Figure 11, the numerical results successfully reproduce the macroscopic brittle response of PC and the enhanced ductility of SFRC. The stage-wise evolution is summarized as follows:

(1) Linear elastic and hardening stages

At the early loading stage, the simulated curves show excellent agreement with the experimental curves. For both the PC and SFRC groups, the numerical responses in the elastic range nearly coincide with the experimental data points, indicating that the numerical skeleton generated from polygonal aggregates captures the initial elastic modulus reliably. In addition, the slightly higher initial stiffness of SFRC is also reflected in the simulations, supporting the effectiveness of the embedded constraint between the solidified fiber representation and the matrix.

(2) Post-peak softening and residual load-carrying stages

After the peak, the load-carrying capacity begins to decrease, and the simulations and experiments exhibit different softening rates. The quantitative assessment is presented below:

PC: The experimental curve exhibits a typical brittle response with a rapid stress drop. The simulation captures this sharp post-peak decline; however, it softens too quickly in the later softening regime.

Quantified error: At a strain of $\varepsilon \approx 2.5\times 10^{-3}$, the experimental residual stress is approximately 18.54 MPa, whereas the numerical prediction is only 9.79 MPa, corresponding to an underestimation of about 47%.

Overall fit: As a result, the PC group exhibits an RMSE of approximately 4.45 MPa over the full curve. This discrepancy is mainly attributed to the two-dimensional idealization, which cannot fully capture three-dimensional aggregate interlock; consequently, frictional resistance along crack surfaces is underestimated.

SFRC: Compared with PC, both the experimental and simulated curves for SFRC display a pronounced post-peak plateau with a much gentler softening response.

Improved agreement: Owing to fiber bridging, the model predicts the post-peak regime with substantially higher accuracy. The full-curve RMSE for SFRC decreases to 1.70 MPa (markedly lower than 4.45 MPa for PC), supporting the reliability of the proposed modeling approach.

Residual strength: At a strain of A, the model predicts a residual stress of 30.59 MPa, which is close to the experimental value (33.80 MPa), with a relative error of only 9.5%. This indicates that the solidified steel-fiber representation introduces additional fracture energy and effectively mitigates the numerical sensitivity associated with excessive matrix softening.

(3) Physical interpretation of the remaining discrepancies

Although the SFRC group shows high agreement overall, the simulated response remains slightly conservative in the late post-failure stage. This behavior is primarily attributed to a dimensional effect: in real specimens, three-dimensional crack surfaces exhibit much higher tortuosity than the idealized two-dimensional crack plane, providing stronger mechanical interlock. In addition, during pullout, some long hooked-end steel fibers may undergo hook anchorage and/or plastic bending, which contributes extra residual resistance in experiments. The current equivalent constitutive model simplifies these complex geometric anchorage effects to some extent, leading to a modest underestimation of the late-stage residual capacity.

**Figure 12:**
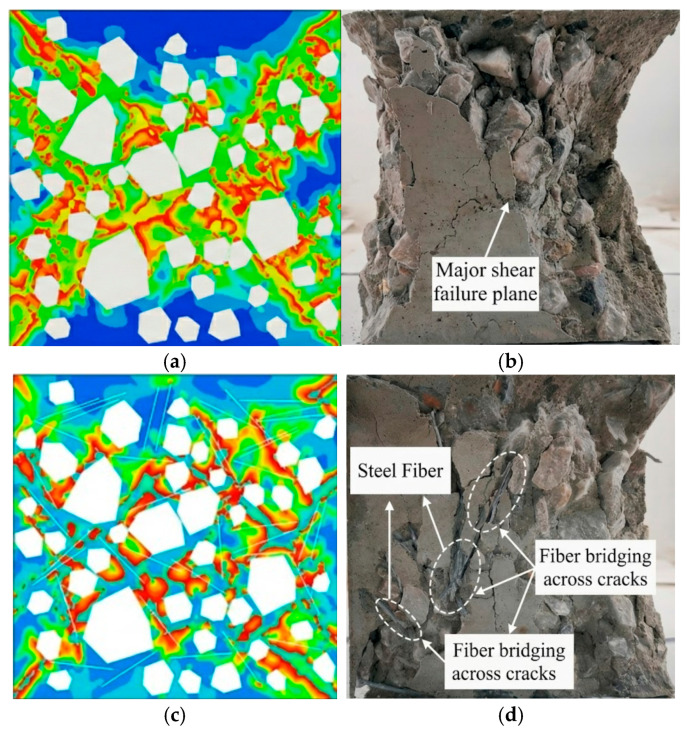
Qualitative comparison of simulated damage localization and experimental failure patterns under uniaxial compression: (**a**) plain concrete (PC), simulation (compressive damage variable, DAMAGEC); (**b**) PC, experiment; (**c**) steel fiber-reinforced concrete (SFRC), simulation (DAMAGEC); (**d**) SFRC, experiment.

Figure 12 provides a qualitative comparison between the simulated damage localization and the experimental failure patterns. For PC, the dominant inclined localization zone in the simulated DAMAGEC contour (Figure 12a) is consistent with the shear-dominated cracking observed experimentally (Figure 12b). For SFRC, the simulated damage field is more dispersed and the formation of a fully connected dominant band is delayed (Figure 12c), which agrees qualitatively with the experimental observation of reduced localization and postponed coalescence due to fiber bridging (Figure 12d).

### 4.3. Limitations and Scope of the Proposed 2D Mesoscale Model

It should be noted that the proposed framework is a two-dimensional plane-strain mesoscale model, and its applicability should be interpreted within this scope. Although the method explicitly improves geometric realism during model generation (especially for fiber volumetric occupation and interference exclusion), it does not fully reproduce three-dimensional features of SFRC failure, such as spatial fiber orientation distribution, out-of-plane crack propagation, and aggregate interlock in three dimensions. These limitations are particularly relevant in the post-peak regime, where 3D crack tortuosity, fiber pull-out path complexity, and aggregate bridging/interlocking may contribute additional energy dissipation.

Therefore, the present model is not intended to replace high-fidelity 3D fracture simulations. Instead, it is designed as an efficient and reproducible mesoscale modeling tool for two-dimensional parametric studies, mechanism-oriented analysis, and comparative numerical evaluation under a unified modeling framework. In this context, the proposed method is expected to provide reliable predictions of macroscopic response trends (e.g., peak strength and relative post-peak evolution) and mechanism-consistent mesoscale damage characteristics, while the quantitative discrepancy in the late post-peak stage should be interpreted as a known consequence of the two-dimensional idealization and equivalent fiber representation.

### 4.4. Practical Implications and Potential Applications

Practical implications of the proposed framework are twofold. From an engineering-design perspective, the batch-reproducible mesoscale models enable systematic parametric studies on fiber content, aspect ratio, and orientation distribution, as well as ITZ weakening levels, thereby supporting performance-oriented optimization of SFRC mixtures and microstructural tailoring. From a computational-workflow perspective, the proposed “skeleton placement–entity generation–topological discrimination–mesh mapping” pipeline avoids repeated manual geometry repair and re-meshing operations that are commonly required in explicit-fiber mesoscale modeling, which improves model-building efficiency and reproducibility. As a result, the framework is particularly suitable for digital testing and comparative evaluation across multiple stochastic realizations, serving as a practical tool for virtual material screening and mechanism-oriented analysis under unified numerical settings.

## 5. Conclusions

This study follows the research thread of “parametric two-dimensional mesoscale configuration modeling of steel fiber-reinforced concrete—solidified interference discrimination—full-curve numerical validation”, and establishes a high-fidelity mesoscale modeling and analysis workflow, which is validated under uniaxial compression. The main conclusions are summarized as follows:

(1) A new mesoscale modeling method for SFRC was proposed based on geometric entity expansion and point–line topology. To address the spurious intersections that can arise from conventional line-based fiber representations and the computational redundancy of circumscribed-circle screening, steel fibers were generated as solidified two-dimensional rectangular rigid bodies. In addition, a vector-cross-product-based Point–Line Method was developed to replace traditional distance-based criteria. The proposed algorithm effectively resolves interference checking for high-aspect-ratio steel fibers in confined spaces, eliminating fiber penetration and mutual interlocking. Moreover, it enables a maximum polygonal aggregate area fraction exceeding 70%, substantially improving both the physical realism and computational efficiency of mesoscale model generation.
(2) A parametric modeling program following the workflow of “skeleton placement–entity generation–topological discrimination–mesh mapping” was developed. Using a Python scripting interface, an automated pipeline was implemented from geometric generation to finite element analysis. The proposed approach exhibits high geometric stability: with identical mix proportions and material parameters, the differences among the stress–strain responses of three random realizations were controlled within 1.5%. In addition, the generated random aggregate models accurately reproduce the initial stiffness of concrete, validating the effectiveness of the polygonal aggregate geometry-correction strategy.
(3) The mesoscale strengthening mechanism of steel fibers in the damage evolution of concrete was elucidated. Damage contour comparisons show that plain concrete (PC) rapidly develops a through-going tangential shear band after the peak load, exhibiting pronounced brittle instability. In contrast, SFRC displays a more diffuse damage pattern, and the coalescence of the dominant crack is significantly delayed. At the mesoscale, fiber bridging between crack faces effectively suppresses damage localization, leading to a clear post-peak “plateau” response and higher residual strength. These observations confirm that the solidified fiber representation can capture the crack-arrest mechanisms associated with fiber bridging.
(4) By comparing the mesoscale simulation results with experimental data for both plain concrete and SFRC, the proposed mesoscale model was shown to reproduce the uniaxial compression response reasonably well, with particularly accurate predictions of the load-carrying capacity of SFRC specimens.

## Figures and Tables

**Table 1:** Key parameters after calibration.

Type	Young’s Modulus/MPa	Poisson’s Ratio	Density/kg·m^−3^	Dilation Angle/°	Viscosity Parameter	Scale Factor *K*	Strength/MPa
Mortar	31,000	0.2	2600	36	0.0005	1.06	50.09
ITZ	27,800	0.2	2600	32	0.0005	1.06	45.32
AGG	43,000	0.23	3500	/	/	/	/
Fiber	210,000	0.3	7850	/	/	/	1150

Note: Mortar and ITZ are modeled using the Concrete Damaged Plasticity (CDP) framework; damage/failure of these phases is represented by tensile and compressive damage evolution (DAMAGET/DAMAGEC) coupled with plasticity. Aggregates are assumed linear elastic inclusions with neither damage nor plasticity. Steel fibers are modeled as embedded T2D2 truss elements with a linear elastic axial constitutive law; the fiber–matrix interaction is represented through the embedded constraint together with the adopted equivalent mapping/bond–slip treatment.

**Table 2:** Comparison table of model data.

Model	Method	Peak Stress/MPa	Peak Strain/10^−3^	Relative Error of Peak Stress/%
Plain Concrete	Exp.	41.51	1.21	0.46
	Sim.	41.32	1.18	
Steel Fiber Reinforced Concrete	Exp.	49.69	1.12	0.31
	Sim.	49.54	1.15	

## Data Availability

The original contributions presented in this study are included in the article. Further inquiries can be directed to the corresponding author.
